# Cranial Bone Changes Induced by Mild Traumatic Brain Injuries: A Neglected Player in Concussion Outcomes?

**DOI:** 10.1089/neur.2023.0025

**Published:** 2023-06-20

**Authors:** Bridgette D. Semple, Olga Panagiotopoulou

**Affiliations:** ^1^ Department of Neuroscience, Monash University, Prahran, Victoria, Australia.; ^2^ Department of Neurology, Alfred Health, Prahran, Victoria, Australia.; ^3^ Department of Medicine (Royal Melbourne Hospital), University of Melbourne, Parkville, Victoria, Australia.; ^4^ Monash Biomedicine Discovery Institute, Department of Anatomy and Developmental Biology, Monash University, Clayton, Victoria, Australia.

**Keywords:** biomechanics, bone, calvarium, fracture, pathology, traumatic brain injury

## Abstract

Mild traumatic brain injuries (TBIs), particularly when repetitive in nature, are increasingly recognized to have a range of significant negative implications for brain health. Much of the ongoing research in the field is focused on the neurological consequences of these injuries and the relationship between TBIs and long-term neurodegenerative conditions such as chronic traumatic encephalopathy and Alzheimer's disease. However, our understanding of the complex relationship between applied mechanical force at impact, brain pathophysiology, and neurological function remains incomplete. Past research has shown that mild TBIs, even below the threshold that results in cranial fracture, induce changes in cranial bone structure and morphology. These structural and physiological changes likely have implications for the transmission of mechanical force into the underlying brain parenchyma. Here, we review this evidence in the context of the current understanding of bone mechanosensitivity and the consequences of TBIs or concussions. We postulate that heterogeneity of the calvarium, including differing bone thickness attributable to past impacts, age, or individual variability, may be a modulator of outcomes after subsequent TBIs. We advocate for greater consideration of cranial responses to TBI in both experimental and computer modeling of impact biomechanics, and raise the hypothesis that calvarial bone thickness represents a novel biomarker of brain injury vulnerability post-TBI.

## Introduction

Mild traumatic brain injury (TBI), including injuries termed concussions, are commonly sustained during activities such as contact sports, assaults, falls, and in military combat.^1^ In children and adolescents, mild TBI (mTBI) accounts for ∼90% of all TBI cases.^2^ The high incidence of sport-related mTBI in youth in particular and the associated health ramifications of these injuries are of great concern internationally.^3^ Whereas a single mTBI may present with relatively benign symptoms and consequences, when TBIs are repetitive, the severity of symptoms typically increases (e.g., headache, fatigue, and memory problems), along with an elevated risk of long-term neurodegeneration and -cognitive deficits.^4–7^ Further, experiencing one mTBI appears to render a person more likely to sustain further TBIs.^8^

To date, research into the effects of single or repetitive mTBI has focused almost exclusively on how these impacts affect the brain itself; while largely neglecting potential involvement of the overlying cranium, which houses and protects the brain. Historically, the cranium has predominantly been studied in the context of severe trauma to the head, in which impact-induced skull fractures contribute to increased mortality and morbidity.^9–11^ Further, though many years of computer simulations have incorporated the cranium in the modeling of brain biomechanics upon impact to the head,^12^ the bone is typically depicted as a simple, inert, and rigid material.^13–15^

However, cranial bone is, in fact, a dynamic and mechanosensitive living tissue, responsive to applied mechanical forces as well as its molecular and cellular microenvironment.^16^ Recent experimental studies have reported that repeated mTBI induces changes in cranial bone composition and structure,^17,18^ whereas other studies have highlighted impact-induced changes in meningeal lymphatic endothelial cells and vasculature as associated with a neuroinflammatory response.^19–22^ These extracerebral effects of an mTBI plausibly have consequences for neuropathology in the associated brain parenchyma and subsequent implications for neurological outcomes for persons with TBI.

We herein review this recent evidence in the context of our current understanding about bone mechanics and the consequences of TBI or concussions on brain structure and function. We build a case suggesting that TBI-induced cranial bone changes may be an important modulator of outcomes after subsequent TBIs, and advocate for greater consideration of cranial responses to TBi in computer modeling.

## Fundamental Concepts

If you cast your mind back to undergraduate neuroanatomy classes, you will recall that the brain is closely enveloped in several protective structures, including the highly cellularized and heterogeneous meningeal layers (the pia mater, arachnoid mater, and dura mater), the cranium, pericranium (periosteum), areolar tissue, *galea aponeurotica*, and skin. The subarachnoid space lies between the pia mater and arachnoid mater and is filled with cerebrospinal fluid (CSF) and vasculature in close association with the lymphatic system.^23^ As a subdivision of the skull (a term that also includes the facial skeleton and the mandible or lower jaw), the cranium is made up of the calvarium enclosing the cranial cavity, which houses the brain. Calvaria consist of large flat bones, such as the frontal, occipital, and the two parietal bones, which are tightly joined together by sutures. Sutures are connective tissues rich in collagenous fibers that increase the elasticity and compliance of the cranium, acting as shock absorbers by absorbing more energy during impact loading compared to the surrounding bone.^24,25^

A TBI or concussion can occur as a result of direct or indirect impact to the head (e.g., blunt or penetrating impact, or a blast shock wave), rapid acceleration, and/or deceleration of the head. Mechanical impacts to the head result in loading regimes (the combination of external forces acting on the head tissues) and deformation regimes (the integral strain and stress regime across the head tissues associated with the loading regime).^26^ These strains and stressors interact with the impacted object (the head). Neither the head nor the cranium are uniform or simple structures, which causes considerable complexity for scientists, clinicians, biomechanical engineers, and physicists attempting to define the impact-deformation relationships of head tissues and understand how and why a given TBI results in neurological consequences. However, put simply, a TBI occurs when the physical load to the head exceeds its capacity to absorb the force without injury to the brain tissue.^27^

## Calvaria Impacts Contribute to Traumatic Brain Injury Pathology

Calvaria fractures caused by blunt-impact TBIs are dependent on the velocity of the impact force and can lead to TBI.^28–33^ The likelihood of calvaria fracture resulting from blunt impact is also dependent upon the geometry and compliance of the bone and the resulting tensile strain.^34^ Thus, the heterogeneous nature of calvarium morphology, varying across anatomical locations, determines differential risk of fracture when blunt impacts are sustained to different aspects of the head.^35–39^ Further, the cranium does not respond to external mechanical stimuli in isolation. Rather, an impact force propagates through the bone and into the underlying, closely associated meninges. These meninges are comprised of heterogeneous cell types with distinct mechanical properties, depending upon variables such as the extent of vascularization and cell density.^40^

At high-impact velocities, calvarial fracture dissipates at least some of the impact energy, which reduces its transfer into the underlying brain tissue. Thus, fractures resulting from high-velocity blunt impacts are likely to reduce the risk of diffuse brain injury, but may increase the risk of brain contusion.^39^ In one study, the evaluation of TBIs resulting from motor vehicle accidents found that the presence of cranial fractures, in fact, lowered the incidence of intracranial lesions, which the researchers surmised to be attributable to reduced intracranial pressure.^41^ However, considerable evidence from both clinical and experimental studies demonstrates that the presence of a calvarial fracture reflects a more severe brain injury and is typically associated with worse outcomes. For example, observational studies in severe TBI patients have found that those with a cranial vault or cranial base fracture have an increased risk of death, whereas the odds ratio of in-hospital mortality was even higher for those who sustain fractures at both sites.^10,11^ In adolescents and adults with TBIs, cranial fracture has been identified as a stand-out significant independent risk factor for the development of intracranial hemorrhage.^9,42^ Further, the risk of developing post-traumatic epilepsy is substantially elevated by an impact-induced cranial fracture (compared to those who sustain a TBI but no fracture).^43^

Animal models have provided some insight into the pathophysiology that results from a calvarial fracture resulting from blunt force impact. For example, in a weight-drop injury model, mice that exhibit a fracture show an exacerbated inflammatory response compared to injured mice without fractures, with the investigators concluding that fractures account for a notable proportion of the variability observed in this model.^44^ Even bone fractures in more remote locations, such as in the limbs, result in worse neuropathological and neurological outcomes after a TBI,^45–47^ indicative of complex bidirectional signaling mechanisms at play beyond isolated consideration of the brain's response to impact force.

The relationship between calvarial trauma and mTBI (i.e., below the threshold of impact that induces a bone fracture) is even less well defined.^48^ This is attributable to factors including ambiguity around the definitions and diagnosis of mTBI and concussion,^32,49^ as well as the involvement of both linear and rotational/acceleration forces associated with concussions. Helmet or mouth guard-mounted impact sensors to collect *in vivo* real-time data on TBIs sustained during participation in contact sports is helping one to better understand the forces required to induce a concussion (e.g., see past works^50–52^). However, even exposure to mTBIs that do not result in known or suspected clinical symptoms (i.e., a “subconcussive” impact) may nonetheless result in physiological, anatomical, and neurological alterations when repetitive in nature.^53,54^ In the next section, we postulate that even if the cranium does not fracture, variation in bone thickness to alter surface area and bone density may reduce the impact strains that result in brain injury, and that such changes may contribute to the biomechanics of mTBI.

## Mild Traumatic Brain Injuries Alter the Calvarial Bone and Underlying Meninges

Much of what we know about the effects of TBIs on the cranium relates to fractures, as described above. However, only a subpopulation of moderate and severe TBI cases are associated with fracture. mTBI, by definition, is typically at a severity below that which results in a fracture, but nonetheless can have serious and long-lasting consequences.^55,56^ A recent systematic review of clinical evidence concluded that the cranium can be deformed even by an mTBI.^57^ Further, several case studies suggest that damage proximal to the cranial base in particular can contribute to neurovascular dysfunction, which may underlie some of the acute symptoms associated with concussion, such as dizziness and headache.^57^

Emerging pre-clinical research has demonstrated that mild impacts to the head, below the bone's optimal strain environment, can also has a notable effect on the calvarium. Using micro computed tomography (CT) and models of repetitive mTBI in rodents, two studies from our group have indicated that mTBIs result in increased cranial thickness.^17,18^ We first reported that a single mTBI to male adolescent mice led to increased calvarial bone volume 5 weeks later.^18^ This increased bone appeared to provide protection against cranial fracture, given that the fracture incidence was considerably lower in this group when subjected to a subsequent mTBI, compared to an age-matched control group who had not received a past mTBI impact.^18^ More recently, we reported that young adult female rats exposed to two or three repeated mTBIs exhibited time- and location-dependent increases in bone thickness and density at the site of impact.^17^ Together, these findings represent a phenomenon of increased cranial thickness after mechanical trauma, which can be observed in different TBI models, species, or injury locations.

Of note, these findings to date are primarily qualitative, with little exploration of the biological mechanisms by which an applied blunt impact force drives an increase in cranial thickness. However, consistent with observations in the context of tibial compression, it is likely that impacts to the cranium induce a local reparative response, including increased bone formation by osteoblasts.^58,59^ Indeed, comparison of cells from load-bearing bones versus non-loaded bones has revealed innate differences in their sensitivity to mechanical loading, leading some researchers to propose that bone cells are either programmed to their physical environment or can adapt and adjust their mechanosensitivity in response to different strain environments.^60^

Further, recent studies have also demonstrated that the meninges mount a dynamic transcriptomic response to mTBI,^20,22^ which is more pronounced in the aging brain, and may contribute to age-related vulnerability and poor neurological outcomes.^22^ mTBI causes changes in meningeal lymphatic vascular morphology and associated impairments in drainage, with some evidence that lymphatic deficits contribute to the exacerbation of TBI-induced inflammation and cognitive dysfunction.^61^ Most recently, new evidence has emerged demonstrating that CSF can access bone marrow niches within the cranium to regulate local immune responses in the context of spinal cord injury and bacterial meningitis.^62,63^ It is plausible that mTBIs also perturb these cranial marrow cavities and their resident immune and hematopoetic stem cells after an mTBI, to influence the effect of the impact on the underlying brain tissue, although empirical evidence of this is currently lacking.

## Individual Variability in Calvarial Thickness

When considering the effects of isolated or repetitive mTBI on the cranium, it is also important to note the inherent population-wide heterogeneity in human cranial structure and morphology, including thickness. For example, thickness of the frontal bone has been reported to range from 3.5 to over 11 mm, alongside individual variability in the ratio of diploë to the outer bone tables and morphological differences evident by histology.^64,65^

Multiple factors can influence calvarial thickness, beginning during early development. Growth of the brain and cranium are dynamic and intimately integrated through common molecular signaling pathways and responsiveness to mechanical forces to ensure an appropriately snug fit.^66,67^ For example, the growing brain generates tensile strain on the developing cranial bones, which promotes bone remodeling to accommodate the expanding neural tissue.^68^ This relationship is well represented by cases of hydrocephalus, in which excess CSF results in abnormal pressure within the cranial vault. If left untreated, this pressure leads to thinning of the bone. Early-life placement of a ventricular shunt can, albeit rarely, instead result in chronic intracranial hypotension, leading to thickening of the calvarium in a condition known as *hyperostosis cranii ex vacuo*.^69-72^ In addition, congenital abnormalities in cranial vault development, such as craniosynostosis (the premature fusing of one or more cranial sutures), can also lead to an abnormal calvarium shape and thickness.^73,74^

In the normally developing skull, the complexity and strength of cranial sutures increases with age, with suture morphology reflecting the loading and deformation regimes applied by growth of the neighboring bones.^75^ Calvarial thickness increases through adolescence to early adulthood, with the greatest increase in regions that are subject to the greatest stress, before a gradual decline with advancing age.^76,77^ The skull undergoes its maximal transformation between infancy and adulthood, in terms of shape, stability (suture elasticity), and bone morphology (e.g., porosity of the diploë layer), with consequences for cranial strength and rigidity.^78^

Individual variability in calvarial thickness is also determined by sex (dependent on the cranial region/bone), stature, body mass index, ancestry, and head size.^65,77,79–83^ Although, historically, calvarial thickness was assessed with the use of calipers, measurements can now be made from CT images, three-dimensional models derived from CT data, and computational models.^77,79^ Adoption of these techniques has allowed for a greater appreciation of population heterogeneity on cranial bone structure and shape.^65^ However, whether this heterogeneity influences the dissipation of the forces resulting from a TBI is unknown.

## Calvarial Thickness as a Moderator of Mild Traumatic Brain Injury?

The above-described evidence indicates that calvarial thickness is dependent upon numerous variables, including age and sex, and suggests that exposure to mTBIs can also moderate calvarial thickness by promoting bone formation. Together, these findings raise the intriguing possibility that differences in calvarial thickness may contribute to a person's risk of (or reslience against) symptomology after repetitive mTBI and influence the extent of brain damage that results from an mTBI.

This hypothesis is plausible based on several considerations. First, from an engineering perspective, increased bone thickness may be a protective mechanism to combat tissue strains and prevent fracture. Increased calvarial thickness results in increased bone strength,^64^ which theoretically limits the transmission of force to the underlying meninges and brain parenchyma. However, the actual consequences of thicker calvarial bone on the extent of brain damage after mTBI, either from isolated or repetitive impacts, warrants further exploration. Changes in cranial thickness may alter load pathways, loading, and deformation regimes into the underlying brain tissue, as well as the direction or diffusion of strain,^64^ which could conversely exacerbate neuropathology.

Further, given that epidemiological studies have identified variables such as age and sex as moderators of mTBI outcomes,^84^ age- and sex-based differences in calvarial thickness may be one of the mechanisms underlying this relationship. For example, because of developmental changes in cranial bone thickness with maturation, the child and adolescent cranium has a lower capacity compared to adult bone to act as a shock absorber for applied mechanical forces. In alignment with this rationale, older mice with thicker parietal bones have been shown to have a reduced risk of cranial fracture from a weight-drop injury model compared to young adult mice with thinner bone,^44^ suggesting that a thicker cranium yields protection against fracture and the associated consequences of this damage.

Further supporting this evidence is an older study by Ruan and Prasad, in which they used an ultrasonic tranducer to obtain high-resolution measurements of frontal bone thickness in human cadavers to inform a finite element model. They reported that a thicker cranium results in a lesser degree of bone deformation upon impact, and concluded that a thicker cranium therefore provides increased protection for the brain. Whereas this finding has implications for resistance to cranial fracture, as noted by the researchers, the influence of differential calvarial thickness on the effects of mTBI for the brain is less clear.

## Research Challenges, Opportunities, and Implications

Research in the field of mTBI is rapidly evolving, and new evidence about vulnerability versus resilience factors has considerable implications for public policy, medicolegal interpretations, education, and healthcare. A more holistic understanding of how extracerebral tissues contribute to brain injury outcomes may represent a missing piece in the puzzle to better predict patient outcomes and design appropriate treatment strategies. Animal models of repetitive mTBI allow for the evaluation of *in vivo* physiological responses to TBIs across a time course^12,18,85,86^; yet, species differences in the anatomy of both the brain and cranium can limit translation of these findings to humans. A popular alternative is to examine human cadavers, providing valuable insights into the response of different bone structures to mechanical impact and the understanding of how loads change as forces pass through the cranium.^64,80,87^

However, these scenarios lack an appreciation for how the transmitted force then enters the living brain. To bridge this gap, computer modeling can prove invaluable to assess the contribution of material properties of the head on the transmission of forces upon impact.^12,14,15,88^ Further, recent work in human volunteers has been evaluating the impact of low levels of acceleration on the living brain and cranium by magnetic resonance imaging, including diffusion tensor imaging and magnetic resonance elastography, to better inform computer modeling.^89^

Ideally, these different approaches should be used in a complementary way. For example, sophisticated and accurate computer modeling of the brain under mechanical force from blunt force impact to the head requires accurate representation of the tissues involved as dynamic structures—including the scalp, cranium, vasculature, meninges, and CSF.^90,91^ Increasing studies have reported on complex heterogeneity in structure and mechanical responsiveness of the meningeal layers^40,92^—findings that also have implications for how traumatic impacts to the head propagate through to the brain itself. Even the brain vasculature has been recognized to contribute to the load-bearing capacity of the brain, with major blood vessels in particular being important predictors of the brain strain resulting from diffuse or rotational impacts to the brain.^91^

It remains to be confirmed whether cranial bone thickness is indeed a modifier of how the force from a blunt force impact to the head leads to an mTBI. If this hypothesis is correct, then it would have considerable implications for the field. Together, such research may lead to more realistic injury prediction, improved risk-mitigation strategies, and the development of improved protective gear.^93^ Cranial thickness determined by CT scan may be a novel predictor of outcomes after repetitive mTBI, whereby a thinner cranium renders a person more vulnerable to worse outcomes after injury. Conversely, identification of persons with a thicker cranium after mTBI impacts (compared to either a baseline pre-injury assessment or population-based reference measurements) may have utility as a novel objective biomarker of past mTBI exposures, with implications for return-to-play decisions for athletes and return-to-combat decisions for military personnel. An improved understanding of the degree to which cranial thickness determines the consequences of mTBI may also have broader implications for medicolegal matters and in forensic medicine (e.g., in the identification of persons exposed to repetitive physical domestic abuse).

## Conclusion

A significant knowledge gap exists in our understanding of the effects of mechanical forces applied to the head at a magnitude below that which induces a cranial fracture. We advocate for increased research in the field toward a more holistic appreciation for how TBIs may influence extracerebral structures and tissues, including the cranium and meninges, and how these interact with the brain parenchyma. Based upon emerging evidence of cranial bone variability, both endogenously and in response to impact, we also propose that animal and computer modeling should consider individual differences in calvarial thickness, and take into account changes in calvarium material properties with age and sex, to ensure that we are appropriately modeling the population at greatest risk of repeated mTBI. The recent generation of reference measurements from the crania of >600 persons, accounting for bone region, sex, age, and ancestry, may prove to be a useful resource for interpreting a person's cranium as being of abnormal thickness.^65^ Yet, further research is needed to empirically demonstrate the potential role of cranial thickness on neurological outcomes after mTBI.

## Transparency, Rigor, and Reproducibility Summary

No original data were generated for the purpose of this review. Study preregistration was not performed because it was not possible to do so in a meaningful way in the context of a review article.

## Abbreviations Used

CSF: cerebrospinal fluid
CT: computed tomography
mTBI: mild traumatic brain injury
TBI: traumatic brain injury
